# Recognition of Fine-Grained Walking Patterns Using a Smartwatch with Deep Attentive Neural Networks

**DOI:** 10.3390/s21196393

**Published:** 2021-09-24

**Authors:** Hyejoo Kim, Hyeon-Joo Kim, Jinyoon Park, Jeh-Kwang Ryu, Seung-Chan Kim

**Affiliations:** 1 Machine Learning Systems Lab., College of Sports Science, Sungkyunkwan University, Suwon 16419, Korea; hyejoo98@g.skku.edu (H.K.); hzoo@g.skku.edu (H.-J.K.); jinyunp@g.skku.edu (J.P.); 2 The Department of Sport Interaction Science (SIS), Sungkyunkwan University, Suwon 16419, Korea; 3 Department of Physical Education, College of Education, Dongguk University, Seoul 04620, Korea

**Keywords:** sequence classification, fine-grained motion classification, human activity recognition, recurrent neural network, attention mechanism, interpretability, gait analysis

## Abstract

Generally, people do various things while walking. For example, people frequently walk while looking at their smartphones. Sometimes we walk differently than usual; for example, when walking on ice or snow, we tend to waddle. Understanding walking patterns could provide users with contextual information tailored to the current situation. To formulate this as a machine-learning problem, we defined 18 different everyday walking styles. Noting that walking strategies significantly affect the spatiotemporal features of hand motions, e.g., the speed and intensity of the swinging arm, we propose a smartwatch-based wearable system that can recognize these predefined walking styles. We developed a wearable system, suitable for use with a commercial smartwatch, that can capture hand motions in the form of multivariate timeseries (MTS) signals. Then, we employed a set of machine learning algorithms, including feature-based and recent deep learning algorithms, to learn the MTS data in a supervised fashion. Experimental results demonstrated that, with recent deep learning algorithms, the proposed approach successfully recognized a variety of walking patterns, using the smartwatch measurements. We analyzed the results with recent attention-based recurrent neural networks to understand the relative contributions of the MTS signals in the classification process.

## 1. Introduction

As wearable devices are gaining popularity, wearable-based human activity recognition (HAR) has attracted increasing attention. Some fundamental functionalities have been adopted by many consumer smartwatches. For example, the device may encourage us to stand up if we sit still for a long time or request an SOS if we fall while alone. With the recent advances in sensors and wearable technologies, many studies have investigated using smartwatches as data-collection equipment [1,2,3,4]. To date, many HAR studies have focused on the coarse-grained classification of human movements, such as walking, running, sitting, and lying, each of which is a distinct activity.

However, in various situations, it is often necessary to recognize fine-grained movements. In some cases, fine-grained classification would make computational experiences contextually aware [5]. For example, differentiating regular walking from inclined walking—walking on steps or on a uniform slope—may be required for the precise calculation of human energy expenditure [6]. In addition, recognition of a slight tremor when walking would make screening processes, such as for Parkinson’s disease [7], more precise.

In a similar context, we focus on the fine-grained classification of walking, which is a fundamental movement that comprises the largest proportion of humans’ daily movements, and propose a system that can recognize predefined walking styles in a supervised manner. To that end, we defined 18 different walking styles, such as regular walking, carrying an umbrella while walking, and looking at a mobile phone while walking., as described in Table 1

Many applications require fine-grained activity recognition; however, achieving a high recognition rate is challenging, because similar movements produce similar signals. A recent work reported that errors occurred when its system classified similar movements that involved walking patterns, e.g., differentiating regular walking from vacuum cleaning [2]. Weiss et al. [4] proposed a system that classifies various everyday activities using a consumer smartwatch. They reported that recognizing similar hand-oriented eating activities, such as eating pasta and soup, was challenging. Kwapisz et al. [8] also proposed a system to classify similar walking activities, including regular walking, and ascending and descending stairs. However, ascending and descending stairs were frequently evaluated as identical movements.

Extensive feature-engineering work may mitigate such recognition issues; however, finding the ideal set of features for a classification process would be time-consuming [9,10]. Thus, classification with manually defined features may not be able to capture subtle differences in similar but different complex temporal patterns. To address the challenges in recognizing fine-grained activities, we adopted recent deep neural networks, such as one-dimensional convolutional neural networks (Conv1D); gated recurrent neural networks (RNNs), such as long short-term memory (LSTM); and gated recurrent units (GRU).

Although deep learning algorithms can learn complex and hierarchical features automatically from raw multivariate timeseries (MTS) data, the learning process is normally not designed to explain how its internal model works. To learn an interpretable representation and visualize the indicators of the raw data that seems influential in the model’s evaluations, we further utilized attention-based neural networks.

The primary contributions of this paper are as follows:

1. We defined a set of fine-grained walking styles that appear every day and proposed a wearable system that can recognize these predefined patterns in a supervised fashion.
2. We conducted an experiment to validate the feasibility of an intelligent wearable system with feature-based machine learning and recent deep learning algorithms, including attention-based deep neural networks.
3. We visualized and analyzed the parameters in the attention layer, which indicate the extent to which the classification result would depend on input signals from different time steps.

## 2. Related Work

### 2.1. Fine-Grained Recognition of Walking Activity

Although quality of walking is used as a measure of the healthiness of a person [7,11,12], few studies have undertaken detailed classification of walking motion, as summarized in Table 2.

In an earlier pioneering work, Bao and Intille [13] proposed a system that classifies daily movements, including activities related to walking, e.g., regular walking, walking while carrying items and ascending stairs, using the measurements from multiple on-body accelerometers. They found that overall recognition accuracy was highest when a decision-tree classifier was used for the task. They also envisioned that machine learning algorithms could be used to recognize different types of walking styles, such as walking slowly and walking briskly. In another study, a smart band-based wearable system was proposed to recognize five different walking styles, such as while texting or calling, with hands in pockets, whilst carrying a suitcase and regular walking, and achieved high and robust classification performance with a support vector machine (SVM)-based classification model [14]. Another previous work proposed a wearable system that utilized gait phase information [15]. Based on the walking distance-estimation algorithm and a decision-tree model, their system successfully recognized three different walking strategies, such as regular walking, walking upstairs and walking downstairs. Interestingly, another previous work demonstrated that acceleration information could be used to recognize differently inclined surfaces in a supervised fashion [16]. They proposed using customized time-frequency domain features to recognize different inclined walking based on a Gaussian-mixture-model classifier. Their experimental results demonstrated its remarkable classification accuracy. They also emphasized that the normalization process for features is crucial to minimize individual variation. A HAR system, based on a body-worn smartphone, was proposed in another recent study [17]. The proposed deep neural network learned the features successfully in an end-to-end fashion, after turning raw input signals into a multi-channel image using Fourier and wavelet transformations, resulting in high classification performance.

Table 2 summaries previous studies on walking-related activity recognition.

### 2.2. Smartwatch-Based Activity Recognition

With the advances in sensor and wearable technologies, studies using smartwatches to recognize human activities have been increasing. In real-life situations, using a smartwatch to capture human activity is advantageous as compared to using a smartphone, in that a smartwatch it is normally placed on a specific body part (e.g., wrist) and does not interfere with body movements.

One crucial but implicit assumption of using a smartwatch to recognize various human activities is that different types of activities would result in different hand movements; thus, types of the whole-body activities could be recognized (or observed) differently using measurements from the smartwatch. Based on this assumption, there have been numerous studies on HAR using recent smartwatches, particularly during the last decade.

For example, an earlier study investigated the possibility of using a smartwatch to recognize 18 different everyday activities [20]. Remarkably, they achieved high accuracy and F*_m_* by proposing a stacked architecture, comprised of a convolution neural network (CNN) and LSTM.

In another previous work, Mekruksavanich et al. [21] proposed a smartwatch-based system that can recognize six different human activities, i.e., sitting, standing, lying, walking, walking upstairs and walking downstairs, in the context of preventing office workers syndrome. With nine different selected features and an ensemble model, they achieved 93.5% classification accuracy. In a follow-up study, they used an LSTM-based deep neural network and achieved 96.3% classification accuracy [22].

A recent work explored and validated the feasibility of sensing hand-oriented activities using consumer smartwatches [5]. Based on an analysis of the spatiotemporal aspect of inertial hand movements using a recent deep CNN model, they achieved 95.2% accuracy across 25 fine-grained everyday hand activities.

Although we have summarized relevant recent studies, it is important to note that research into smartwatch-based activity recognition systems is in an early stage.

**Figure 1:**
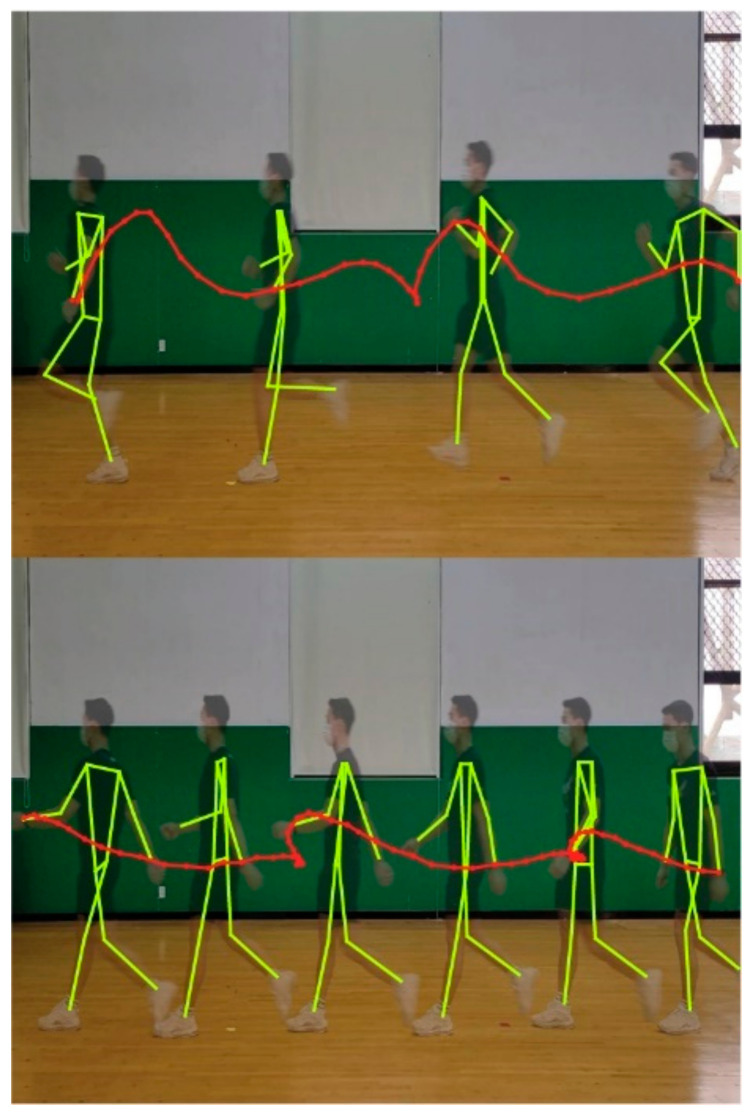
An example of the different hand motions affected by walking strategies: running (**upper**) and regular walking (**bottom**). The position of the left hand is highlighted and connected by a dotted line. Note that walking strategies significantly affect the hand motions while walking (e.g., spatiotemporal walking parameters, such as the speed and intensity of the waving arm). Here, the poses overlayed on the pictures have been extracted based on a recent work [23].

In this paper, we assumed that different types of walking activities generally involve different dynamic hand motions, as shown in Figure 1. Note that different walking strategies would result in different arm-swing patterns. From this perspective, we hypothesized that differences in MTS motion signals from different walking patterns could be learned by machine learning algorithms. To validate our hypothesis, we first developed an intelligent wearable system that leverages recent advances in artificial neural networks. Then, we conducted an experiment in which participants were asked to walk as instructed with the device on their wrist. We will describe the experiment and the results in the following section.

## 3. Experiment

In this section, we first describe the wearable system developed for the proposed fine-grained activity recognition task. As described in the previous section, we focused on the wrist-worn smartwatch as walking patterns affect the hand motions while walking, differently from the previous studies that focused on the sensors attached to the leg [15] for recognizing the walking patterns. We then describe the activities defined in this study and the experimental procedure conducted to validate the proposed approach’s feasibility.

### 3.1. Equipment

**Figure 2:**
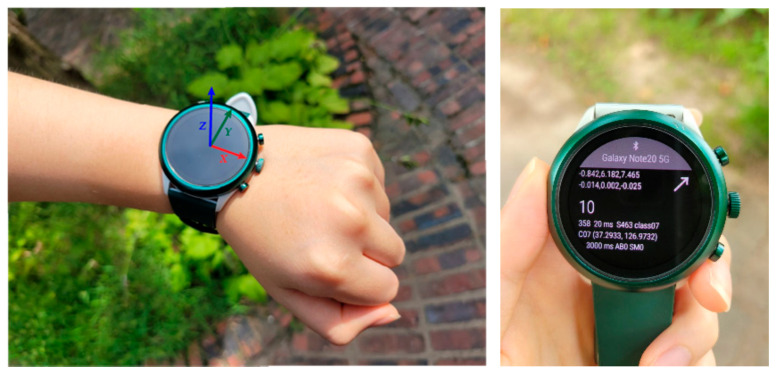
Smartwatch system with its axes displayed (**left**), and a screen from the running application created to capture dynamic hand motion (**right**).

In the data collection process, we used a consumer smartwatch (DW9F1 by Fossil Group, Inc., Texas, USA) as the sensing device and a smartphone (Galaxy Note 20 by Samsung Electronics Co. Ltd., Korea) as the host device. For the smartwatch, we developed a custom application to capture the inertial movements of the hand in the form of MTS data using Wear OS by Google. Here, sensor values from the built-in motion sensors (e.g., triaxial accelerometer and gyroscope) were captured at every 20 ms. For the smartphone, we developed a custom host application to manage smartwatch application remotely over the Bluetooth low-energy (BLE) connection. With the host application, the experimenter can assign a label to the motion, take notes for the experiment, and control the start and end of the capture process remotely. Figure 2 shows the smartwatch device used in this study (left) and an example of the custom application running on the smartwatch (right).

### 3.2. Activity Definition

**Figure 3:**
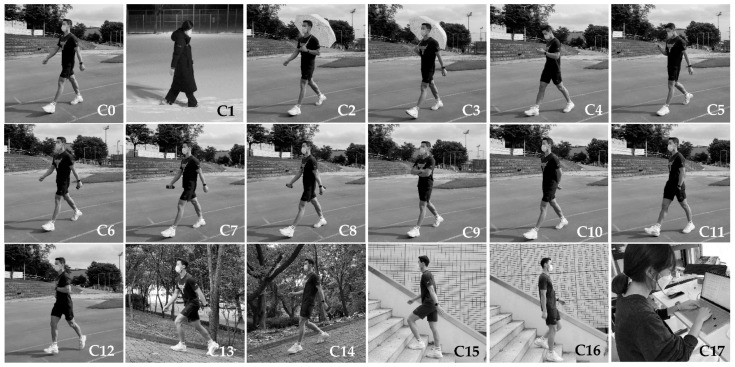
Example photographs taken to describe the 18 different walking styles defined in Table 1. Class index is displayed at the bottom-right corner of each picture. Class #C17 (i.e., doing something while sitting and standing) was added as the reference class.

We defined a set of 18 different walking styles (Table 1 and Figure 3) that are used frequently in daily life. For motion classes C4 and C5, we asked the participants to read arbitrary content displayed on the smartphone while walking. For motion classes C6, C7, and C8, the participants walked while holding a 2-kg dumbbell (approximately 4.4 pounds) in the left, right, and both hands, respectively, to simulate holding a heavy load (e.g., groceries).

### 3.3. Proposed Method

#### 3.3.1. Problem Definition

**Figure 4:**
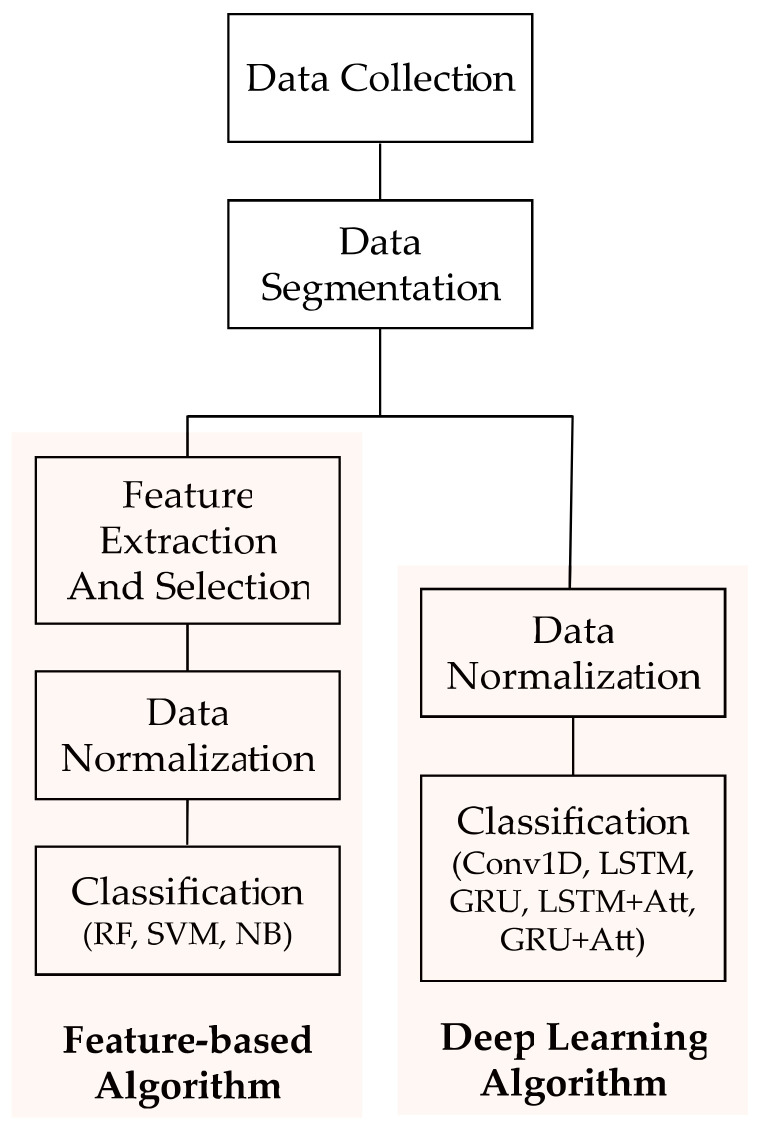
Pipeline of the machine learning process. A feature-based approach, in which machine learning step is preceded by a feature engineering process is used as the baseline models of deep learning-based approach.

Given the MTS input data $x=\left(x^{\langle 1\rangle },x^{\langle 2\rangle },\ldots ,x^{\langle T\rangle }\right)\in {\mathbb{R}}^{T\times D}$, the machine learning systems for activity recognition attempt to estimate $y\in {\mathbb{R}}^{M}$, i.e., a type of activity from a predefined set of activities. Here, $x^{\langle t\rangle }\in {\mathbb{R}}^{D}$ represents the *t*-th measurements, *T* and *D* (=6 in our case) represent the length of the signal and the dimension of the sensor data, respectively, and *M* denotes the number of activity types. Figure 4 shows the pipeline of the machine learning process used in this study.

#### 3.3.2. Data Collection

Thirty-six subjects (20 to 62 years old; average age: 27.91; standard deviation: 11.57 years) participated in this experiment. Note that all participants self-reported being right-handed. In this experiment, the participants wore the smartwatch on their non-dominant hand (i.e., the left wrist).

The participants were asked to walk according to the instructed walking styles. For class C0, we instructed participants to walk at a self-paced speed but not at higher intensities exceeding moderate levels. Most participants walked at least one lap around the 400-m campus track.

For classes C15 and C16, the participants were moved to stairs, and for classes C13 and C14, the participants walked up and down ramps (inclined approximately 10 degrees), respectively, on the university campus.

Although the experiment was conducted in different seasons (winter to summer), the amount of data obtained for classes C2 and C3 (walking with an umbrella) and class C1 (walking on thick snow) was relatively small compared to the other cases because specific weather conditions were required for data collection. In addition, a relatively small amount of data was collected for class C12 (jogging) because this task was performed in a shorter time over the same distance. Note that we instructed the participants to stop the trial whenever they felt uncomfortable, to avoid becoming tired after the experiment.

The total time taken for each class is shown in Table 3. Cumulatively, we collected a total of 45.18 h (std: 0.72) of data from the 36 participants.

#### 3.3.3. Data Segmentation

**Figure 5:**
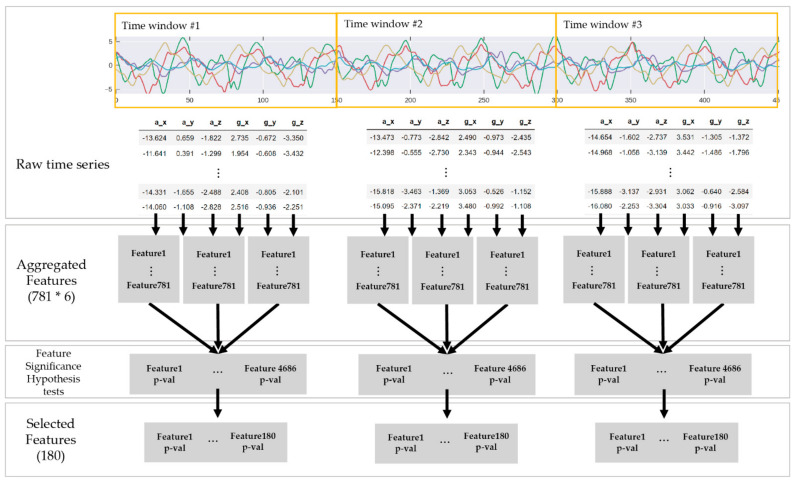
Flow of the feature-extraction and -selection processes using the tsfresh library [9]. Final feature set is selected according to the *p*-values from the feature significant test.

As described in Section 3.1, labelled information was assigned by the host device during the experiment. The collected MTS data were normalized by removing the mean and scaling to unit variance on each axis. The preprocessed data were then segmented using two different partitioning windows (*T* = 100 and 150 samples, accounting for 2 and 3 s of movement, respectively) without overlaps between adjacent segmentations. Here, we selected a motion segment length of *T* = 100 and 150 because common walking activities have a cycle of less than 2–3 s. Note that we did not align the signals according to the walking phase so that the machine learning models could learn features from each activity regardless of the activity phase (Figure 5), a viable strategy according to a recent study [17].

#### 3.3.4. Feature-Based Machine Learning

Rather than relying on time-consuming feature-selection tasks, we employed the tsfresh library [9] to extract statistically significant timeseries features. The tsfresh library provides highly parallel feature selection algorithms based on the Benjamini–Yekutieli procedure [24], which is a false-discovery-rate-controlling procedure.

In the feature-extraction process, a comprehensive number of features (e.g., 4686 = 781 × 6 features in our case) was extracted from the segmented MTS signal $x\in {\mathbb{R}}^{T\times D}$. We then selected the 180 most-significant features. Here, approximately 30 features could be extracted for each axis based on the significance hypothesis test. The entire feature extraction process is illustrated in Figure 5, and Table 4 shows the 12 most significant features based on the results of the feature significance hypothesis test.

As the baseline, we used a set of feature-based classifiers, including naïve Bayes (NB), support-vector-machine (SVM) [25], and random-forest (RF) [26] classifiers. The NB classifier is a probabilistic model based on Bayes’ theorem [27]. The NB classifier is applicable to many practical problems; however, its performance often degrades due to the naïve assumption that features are conditionally independent and contribute equally to the output. The RF classifier utilizes ensemble learning, which is a machine-learning technique that combines many decision-tree classifiers. The RF classifier can handle high-dimensional data efficiently and can mitigate the overfitting issue [28]. The SVM classifier is a machine-learning tool that is effective at classifying high-dimensional data [25]. In this study, the radial basis function (RBF) was used as the kernel function.

#### 3.3.5. Deep Learning Algorithm

We adopted Conv1D, LSTM, and GRU to learn features and classify the segmented MTS signal $x\in {\mathbb{R}}^{T\times D}$. In addition, we employed attention-based LSTM and GRUs to learn an interpretable representation that describes which parts of the input sequence receive the model’s attention during classification. We adopted the attention mechanism, initially devised for machine translation tasks, for densely visualizing the machine attention to explain and interpret how the models come to a decision.

##### Conv1D

A convolutional neural network (ConvNet) is a particular kind type of artificial neural network comprised of multiple building blocks, e.g., alternating convolution and pooling layers to learn features, and fully-connected layers for classification and regression. A ConvNet extracts local features efficiently at a specific hidden layer by limiting the size of the receptive fields of filters (i.e., sparse connectivity). It also learns the spatial hierarchies of features using stacked deep-layer structures. Especially during the last few years, it has successfully demonstrated its capability to learn features from different types of information, such as regular image, spectral data [5,17,29], 3D volumes [30], etc. In a one-dimensional convolutional neural network (Conv1D), convolutional kernels are convolved with the layer input over a single temporal/spatial dimension [31,32] to produce latent features. Conv1D can learn hierarchical features with low computational complexity, as the major operation is a simply weighted sum of two one-dimensional arrays [33], it is widely used in many practical sequence classification tasks, e.g., sentence classification [32], earthquake detection [34], surface recognition [35], context understanding [36], and real-time electrocardiogram monitoring [37]. Similar to a recent work [38], we set all the kernel sizes (i.e., the length of the 1D convolution window) as 3 and the stride length of the convolution as 1.

##### LSTM

The standard RNN with the traditional *tanh* unit suffers from the vanishing and exploding gradient problem, which makes difficult its learning long-term dependencies. LSTM was proposed to mitigate this issue. LSTM can learn long-term dependencies using memory-cell and gate units [39], and LSTM-based architectures have been employed in many sequence classification applications [35,36]. The memory cell stores information taken from the input and previous cells over the given period. This information is controlled by the gate units, i.e., update, forget, and output gates.

##### GRU

Similar to LSTM, the GRU [40] performs better than the basic RNN in many sequence transduction tasks, e.g., language modelling [41], torque generation [42], and many sequence classification tasks [36,43]. For the GRU- and LSTM-based architectures, we stacked recurrent cells two times (i.e., stacked two-layer GRU/LSTM [44]) to retain more long-term dependence information. The dimensionality of the output space of the recurrent hidden states was set to *T*, identical to the length of the input signal $x\in {\mathbb{R}}^{T\times D}$.

##### GRU and LSTM with Attention Mechanism

**Figure 6:**
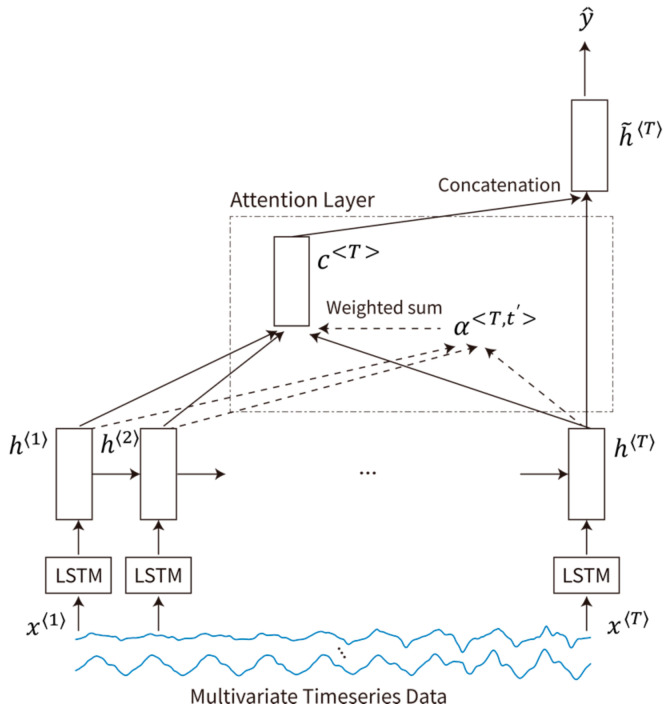
Multiplicative attention-based LSTM/GRU for the classification process.

Although gated RNNs, e.g., LSTM and GRU, and Conv1D have demonstrated their effectiveness in various sequence classification tasks, it remains difficult to explain and interpretate how the models come to a decision. Thus, for the proposed classification task, we utilized attention-based RNNs, which are typically applied to a variety of sequence transduction tasks in which alignments between different modalities must be learned [45,46,47]. Here, we adopted a multiplicative attention mechanism, which reduces encoder/decoder states to an attention score via a simple matrix multiplication [46]. As shown in Figure 6, our network comprises an LSTM/GRU-based sequence encoder, an attention layer, and a classification layer.

Given the MTS input data $x=\left(x^{\langle 1\rangle },x^{\langle 2\rangle },\ldots ,x^{\langle T\rangle }\right)$, where $x^{\langle t\rangle }\in {\mathbb{R}}^{D}$ represents the *t*-th measurement, the sequence encoder generates a sequence of hidden states $a=\left(a^{\langle 1\rangle },a^{\langle 2\rangle },\ldots ,a^{\langle T\rangle }\right)$, where $a^{\langle t\rangle }\in {\mathbb{R}}^{h}$ represents the output of the *t*-th data point.

The context vector, which is a weighted sum of a and captures relevant source-side information to predict the label of the input signal, is calculated by multiplying attention weights α with the encoder outputs a as follows.

(1) $$c^{\langle T\rangle }=\sum_{t^{\prime }}\alpha^{\langle T,t^{\prime }\rangle }a^{\langle t^{\prime }\rangle }$$

Here, $\alpha^{\langle T,t^{\prime }\rangle }$ describes the amount of attention that ${\hat{y}}^{\langle T\rangle }$ should pay to the input feature at time $t^{\prime }$ (i.e., $a^{\langle t^{\prime }\rangle }$ ). As shown below, the alignment score is normalized with a softmax layer to produce the attention weights.

(2) $$\begin{array}{c} \alpha^{\langle T,t^{\prime }\rangle }=\mathrm{softmax}\left(\mathrm{score}{\left(a^{\langle T\rangle },a^{\langle t^{\prime }\rangle }\right)}_{t^{\prime }=1}^{T}\right)\\ =\frac{\exp\left(a^{\langle T\rangle }W_{a}a^{\langle t^{\prime }\rangle }{}^{⊤}\right)}{\sum_{t^{\prime }=1}^{T_{x}}\exp\left(a^{\langle T\rangle }a^{\langle t^{\prime }\rangle }{}^{⊤}\right)} \end{array}$$

Here, $\mathrm{score}\left(\cdot \right)$ is a bilinear function, which compares the two hidden states, and $W_{a}$ is the trainable weight matrix of attention. The length of the alignment score α is T. Differing from attentional encoder–decoder problems [45,46], in our classification problem, $a^{\langle T\rangle }$ is the last hidden state of the *encoder* network because our problem does not involve any decoder structure. A similar approach was used in recent studies [48,49].

The attentional hidden state ${\tilde{h}}^{\langle T\rangle }$ is produced by concatenating the context vector $c^{\langle T\rangle }$ and the last hidden state $a^{T}$ as follows:

(3) $${\tilde{h}}^{\langle T\rangle }=tanh\left(W_{c}\left[c^{\langle T\rangle };a^{\langle T\rangle }\right]\right).$$

Then, the attentional vector ${\tilde{h}}^{\langle T\rangle }$ is used to calculate the probability and label of the output ${\hat{y}}^{\langle T\rangle }$ as follows.

(4) $$p(y|x)=softmax\left(W_{s}{\tilde{h}}^{\langle T\rangle }\right).$$

(5) $${\hat{y}}^{\langle T\rangle }=argmax_{y}\;p(y|x)$$

For the cost function of all deep learning-based approaches, we employed cross entropy between measured values, y, and estimated values, $\hat{y}$, which is defined as follows:

(6) $$CE=-\sum_{i}^{m}y^{\left(i\right)}log\left({\hat{y}}^{\left(i\right)}\right)+\left(1-y^{\left(i\right)}\right)log\left(1-{\hat{y}}^{\left(i\right)}\right)$$

where *m* is the batch size. Also, we added a dropout layer to the hidden layer output of all the deep networks to prevent overfitting. The Adam optimizer (with a learning rate of lr = $10^{-3}$, β1 = 0.9, β2 = 0.999) is used to train all of the deep learning-based models outlined to minimize cross-entropy loss [50].

## 4. Results

### 4.1. Classification Results

We use F1 score in the evaluation of each class, defined as harmonic average of precision (*P*) and recall (*R*), and weighted F1 score as the primary performance metric.

(7) $$F1\;=\;\frac{2\cdot P\cdot R}{P+R}$$

To compute mean F1 score (F*_m_*), we weight the per-class F1 scores by the number of instances for each class.

(8) $$F_{m}=2\sum_{c=1}^{C}\frac{N_{c}}{N_{tot}}\;\frac{P_{c}\times R_{c}}{P_{c}+R_{c}}$$

**Figure 7:**
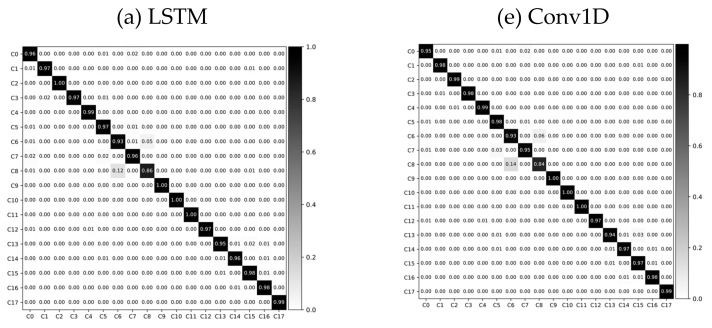

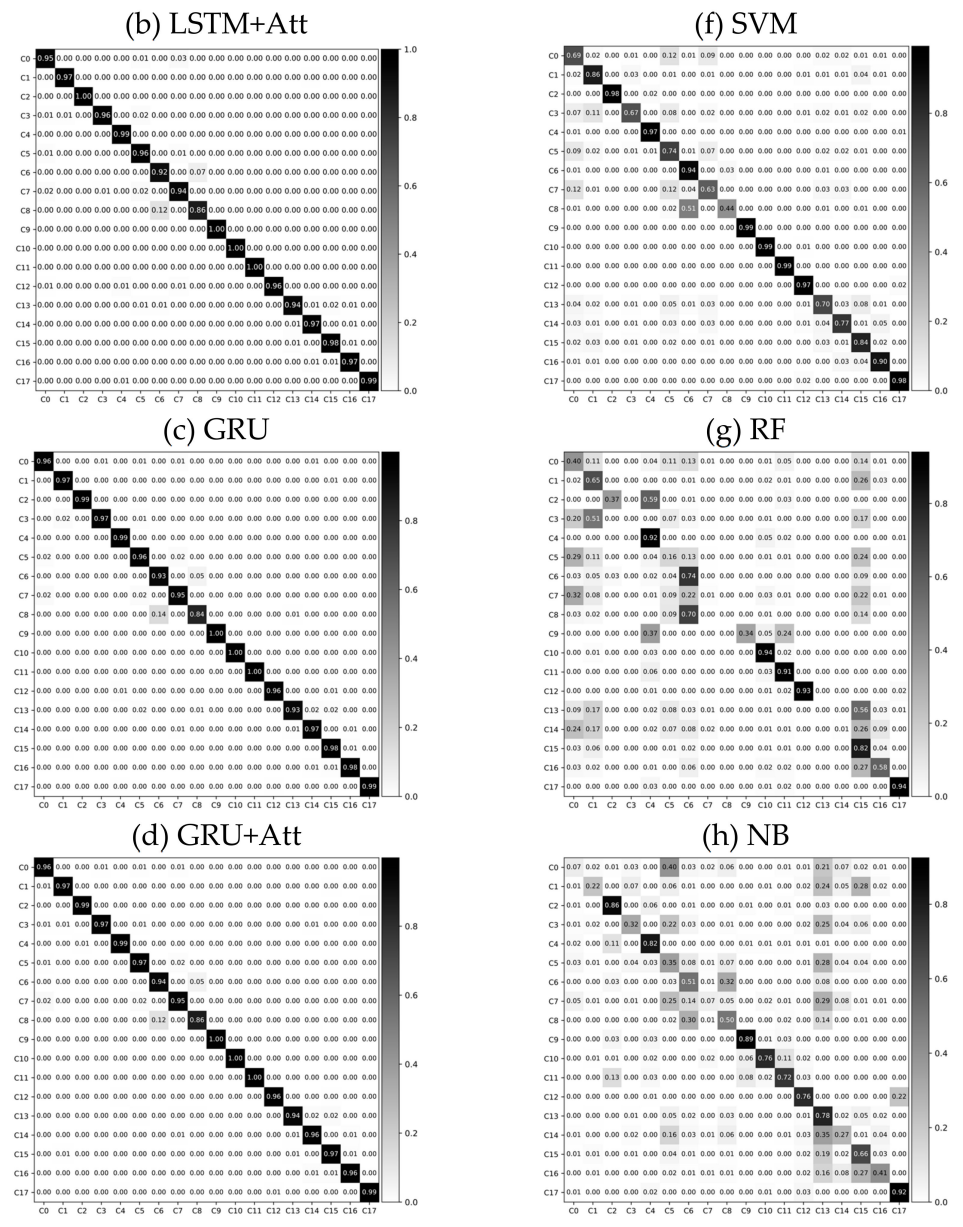
Normalized confusion matrix.

Here, $N_{c}$ is the number of samples that belong to class *c*, and $N_{tot}$ is the total number of the samples from C different classes. Table 5 shows the classification accuracies and F*_m_* obtained from the experiments, and Table 6 shows the mean and standard deviation time required for inferencing a single data sample $\in {\mathbb{R}}^{T\times D}$. Confusion matrices of the results from feature-based and deep-learning algorithms are shown in Figure 7.

For the feature-based learning conducted as the baseline, the accuracy (F*_m_*; weighted F1 score) for SVM, NB, and RF were 84.933 (84.706), 53.382 (52.108), and 53.400 (47.439), respectively. Note, here the length of the partitioning windows was *T* = 150 (approx. 3 s). Despite the extensive feature engineering work, the deep learning-based approach demonstrated higher performance. The accuracy (F*_m_*) for LSTM, LSTM with attention mechanism (LSTM + Att), Conv1D, GRU with attention mechanism (GRU + Att) and GRU when the length of partitioning windows was *T* = 150 (approx. 3 s) were 97.158 (97.156), 97.096 (97.097), 96.976 (96.971), 96.902 (96.903), and 96.788 (96.782) respectively. There was no significant performance difference with the addition of attention. The benefits of an attention mechanism will be discussed in Section 5. Detailed classification performances are listed in Table 5.

**Figure 8:**
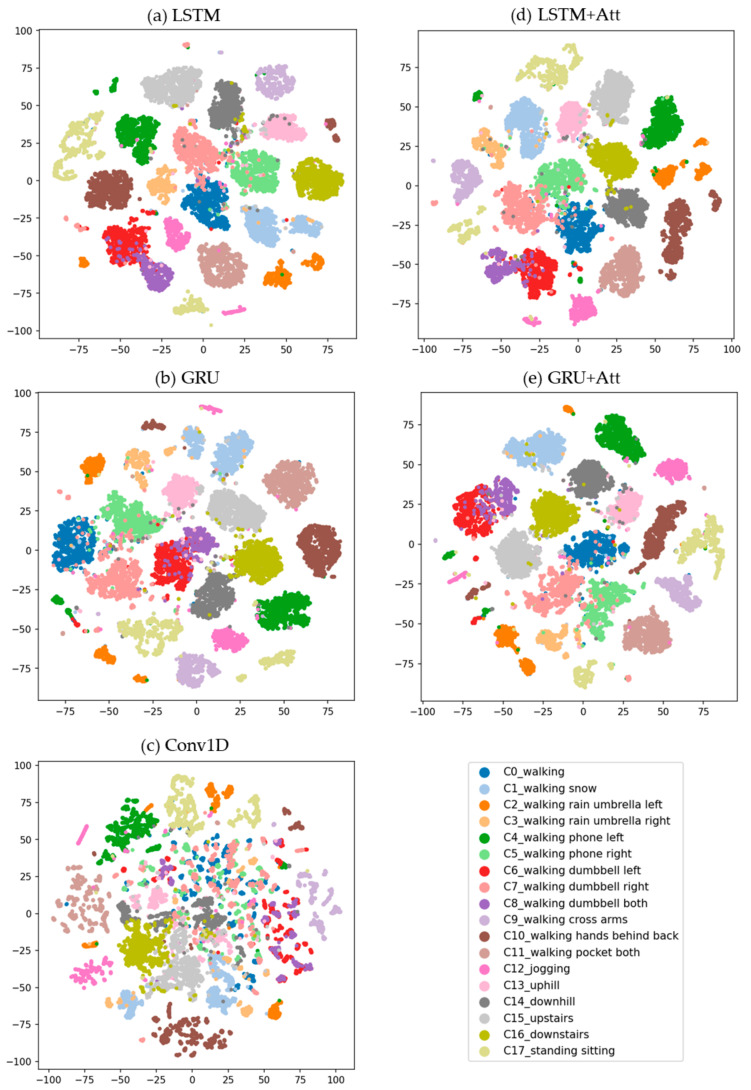
t-SNE visualization of high-dimensional (*D* = 64) internal features of deep-learning-algorithm models, each of which is displayed in the title of the subfigures. Each two-dimensional point represents a segmented motion of *T* = 150 that is projected from the 64-dimensional feature space.

We also examined the high-dimensional internal features (*D* = 64 in our case) learned by our deep neural networks, such as Conv1D, LSTM, GRU, LSTM + Att, and GRU + Att, using t-distributed stochastic neighbor embedding (t-SNE) [51]. The two-dimensional embeddings projected from the last fully-connected layer are shown in Figure 8.

### 4.2. Blind Test

We collected an additional blind test dataset to further evaluate the robustness of the proposed system. The blind test data was collected from the subjects who did not participate in the experiment. We obtained the blind test dataset in a comparable but not identical environment to the training data because the blind test dataset was obtained assuming real-world conditions (e.g., flat walkway and field tracks on campus). Table 7 shows the total time spent on each class. Cumulatively, we collected a total of 35.90 min of data for the 18 classes, which is approximately 1.99 min (std: 0.87 min) for each class. The sampling rate was set to 50 Hz, the same as for the training dataset.

**Figure 9:**
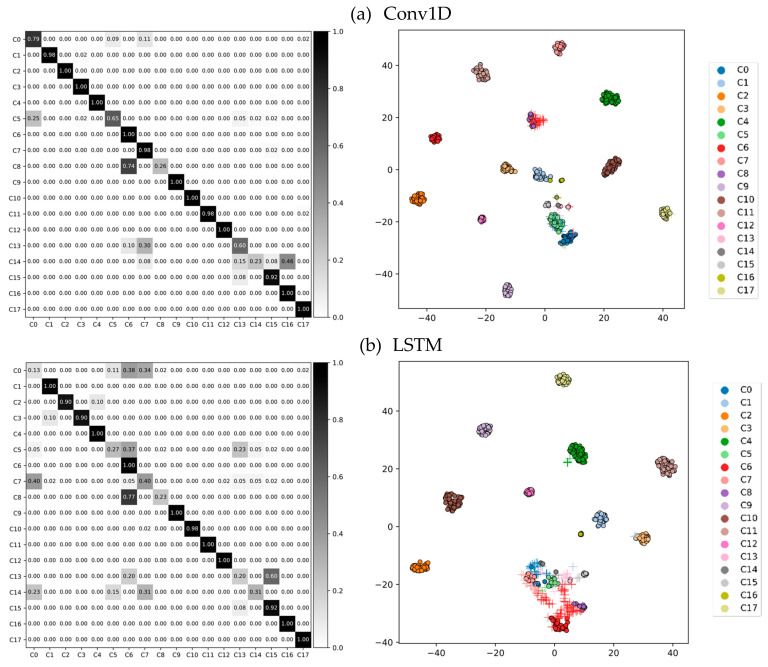
Example of confusion-matrix (**left**) and t-SNE visualization (**right**) of the blind test results when (**a**) Conv1D and (**b**) LSTM-based classifiers were employed. Here, *T* was set to 150. As the t-SNE plot from the Conv1D model shows well-clustered distributions, our model could collect similar activities into similar points in the high-dimensional feature space (*D* = 64). However, results from LSTM demonstrated low accuracy during the blind test.

In the blind test, we analyzed the results using only deep learning algorithms that outperformed feature-based algorithms during the main experiment. The accuracy (F*_m_*) for Conv1D, LSTM+Att, GRU, GRU+Att and LSTM, when the length of partitioning windows was *T* = 150 (approx. 3 s), were 87.290 (88.259), 77.793 (78.167), 74.720 (76.570), 74.022 (75.877), and 74.441 (75.326), respectively. While the LSTM-based classifier demonstrated the best results for the test dataset, Conv1D showed the best classification performance for the blind test dataset. However, compared to the test dataset, the overall Conv1D classification performance, in terms of accuracy and F*_m,_* during the blind test was worse. For example, these values were particularly reduced when recognizing C0 (p: 71.154, r: 78.723), C5 (p: 90.698, r: 65.0), C8 (p: 1.0, r: 26.316), C13 (p: 50.0, r: 60.0), and C14 (p: 75.0, r: 23.077) when Conv1D was employed, where p is precision and r is recall. In case of LSTM, these values were reduced significantly in recognizing C0 (p: 20.690, r: 12.766), C5 (p: 69.565, r: 26.667), C7 (p: 44.737, r: 39.535), C8 (p: 86.667, r: 22.807), C13 (p: 10.526, r: 20.0), and C14 (p: 44.444, r: 30.769). Note that, compared to the results from the LSTM model, the projected feature points in the same class are well clustered together when the Conv1D model was employed, as shown in Figure 9. This may be partly because the pre-trained LSTM model was overfitted to the training dataset.

Figure 9 shows the confusion matrix (left) and the corresponding t-SNE visualization of the blind test set using the Conv1D (upper) and LSTM (bottom) model as a classifier (right). Correctly classified data is marked with a filled circle and incorrectly classified data is marked with a cross.

## 5. Discussion

### 5.1. Classification Performance

In general, based on the overall classification results, deep learning-based approaches successfully learned features from the different fine-grained walking styles defined in our study. During the test phase, it is noticeable that the LSTM/GRU-based approach demonstrated the highest accuracies and F*_m_*, i.e., greater than 96%, in both segmentation conditions. In our study, the addition of an attention layer did not significantly affect classification performance. Conv1D also exhibited high accuracies and F*_m_* over 96% when the length of the segmentation window was *T* = 150 (approx. 3 s). The most challenging activity to recognize was C8 (p: 88.027, r: 86.442) when LSTM was utilized.

In contrast, feature-based approaches demonstrated lower classification performances over almost all the activities despite of the extensive feature-engineering process. Therefore, except for the SVM, it is apparent that the feature-based machine-learning models adopted in our study do not have sufficient capacity for learning the features from proposed fine-grained motion dataset.

Regarding the blind test described in Section 4.2., the accuracy (F*_m_*) was significantly reduced by 9.686 (8.712) percent in the case of Conv1D compared to those from the test dataset. Although our approach validated the feasibility of the proposed learning scheme, robust recognition of some classes, such as C5, C8, C13, and C14, was found to be challenging as shown in Figure 9. More specifically, we found that C5 (walking phone right) was misclassified as C0 (regular walking) when the Conv1D model was used. This may be because there were differences in the degree to which participants focus on their smartphones, although they were asked to read the arbitrary contents displayed while walking. Also, we found that walking with a dumbbell in both hands (C8) was confused with walking with a dumbbell in the left-hand (C6). In addition, walking uphill (C13) was somehow confused with walking with a dumbbell in the right-hand (C7) and walking downhill (C14) was confused with walking downstairs (C16). An earlier work [8] reported a similar misclassification issue: ascending and descending stairs were frequently evaluated as identical movements.

Noting that walking with something in the right hand and walking on inclined/stepped surfaces were successfully recognized in the training and test datasets but not in the blind test dataset, we plan to collect more data on these activities from diverse users to make our model more robust.

Except for these classes, the rest of the classes’ classification performance was better than or similar to the test dataset results. The blind test dataset, on the other hand, was analyzed using a modest amount of data. As a result, additional research with data from the various distributions is required.

### 5.2. Attention Mechanism

Learning an interpretable representation is crucial in many machine-learning tasks. A deep learning algorithm has an advantage of extracting features from the raw data; however, typically, understanding the relative contributions of the input data is a challenging task. To mitigate this issue, the concept of attention was introduced in earlier studies [45,46]. In this paper, we incorporate an attention mechanism, originally devised for neural–machine-translation tasks [46], into our classification model to learn an interpretable representation that describes which parts of the input data are receiving the model’s attention. Different from recent studies on attention-based HAR systems [52,53,54], we further focus on densely visualizing and analyzing the attention weights along with the raw sensor input signal, $x\in {\mathbb{R}}^{T\times D}$.

**Figure 10:**
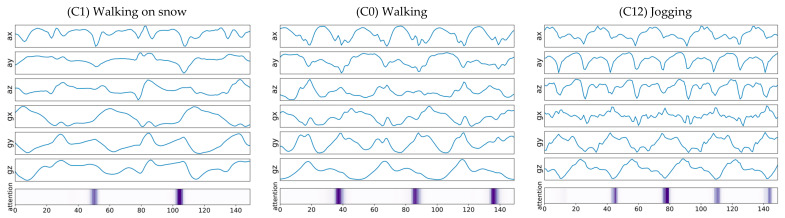
Example of MTS input signals from three different walking activities with temporally aligned attention vectors highlighted. The darker the highlighted bar, the more attention it received from the model, and thus contributing more during the inference phase. If the repetition cycle of the exercise was long, this example indicates that attention peaks shown in purple were formed at a slow cycle.

**Figure 11:**
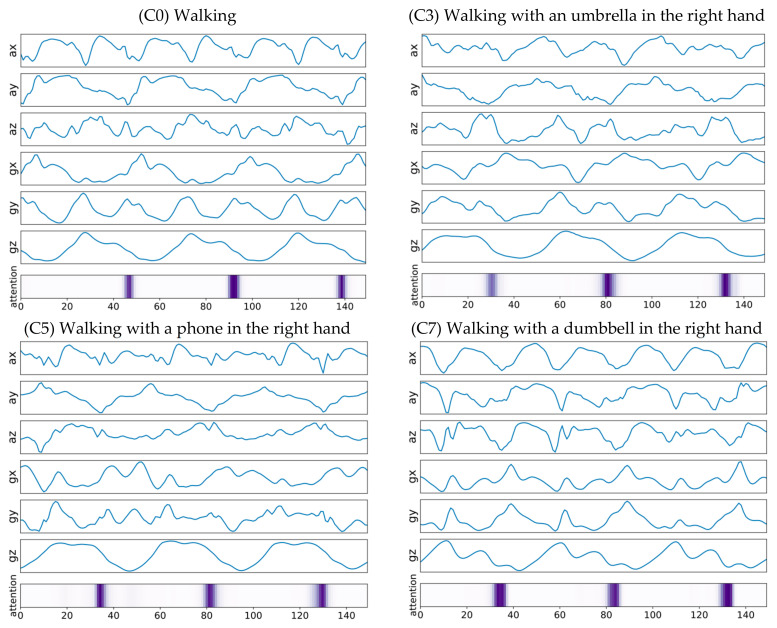
Examples of input signals from walking with something in the right hand, with temporally aligned attention vectors highlighted.

Figure 10 and Figure 11 are examples of visualization of attention vectors, $\alpha \;\in {\mathbb{R}}^{T}$, highlighted in the bottom of each figure. The darker the highlighted bar, the more attention the attention vector received from the model during the inference phase. Note that attention values are formed in a continuous manner. In other words, a machine-learning model takes a collection of adjacent parts of input signals, rather than discrete parts of the signals, during the training and inference phases. This may be because input signals from specific intervals contribute to the calculation of the context vector, which captures relevant source-side information required to predict the label of the given MTS input signals.

### 5.3. Walking with Something in the Right Hand

Activities with something in the left-hand are relatively easy to recognize in that sensor values are recorded in the smartwatch worn in the left hand. There was little confusion reported between walking with an umbrella in the left and right hand (C2/C3) and between walking with a phone in the left and right hands (C4/C5).

Although we initially assumed that it would be challenging to recognize cases in which the objects are being held in the right hand, it turned out that the proposed system could successfully recognize these activities, i.e., walking with an umbrella in the right hand (C3), walking with a phone in the right hand (C5), and walking with a dumbbell in the right hand (C7). This may be because our whole-body motion, including that of the left hand, is somehow affected by the constraints imposed on the right hand. For example, holding an umbrella or a heavy load in the either hand normally affects our dynamic walking patterns, such as spatiotemporal-stride and arm-swing parameters, significantly.

**Figure 12:**
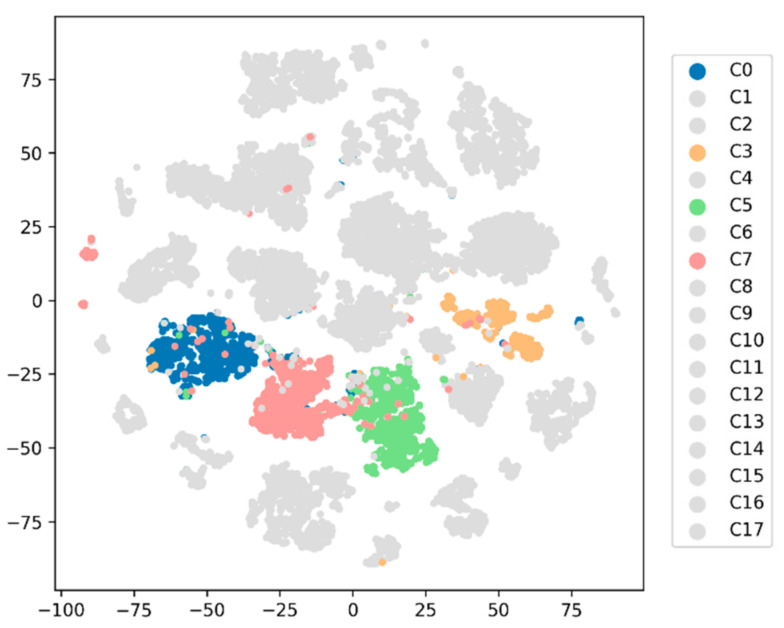
t-SNE visualization of high-dimensional internal features. Only samples from asymmetric activities are highlighted in color. Here, LSTM+Att was employed as a classifier.

Figure 11 shows examples of input signals from walking with something in the right hand (C3/C5/C7) with temporally aligned attention vectors highlighted. Note that the darker the highlighted bar, the more attention it received from the model; thus, contributing more during the inference phase. As shown in Figure 12, the two-dimensional feature embeddings from these activities (C3/C5/C7) are well clustered in distribution and separated those from other types of activities, including regular walking (C0).

**Figure 13:**
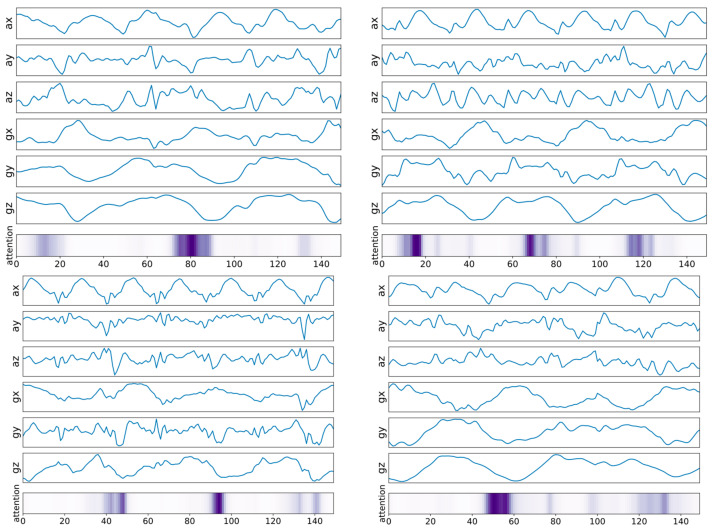
Examples of input signals from C8, each of which was mistakenly recognized as C6, with temporally aligned attention vectors highlighted.

However, as we said in Section 5.1, our system is unable to detect all walking behaviors during the blind test. C8 (p: 100.0/86.667, r: 26.316/22.807 when Conv1D/LSTM were used) was, for example, mistaken with C6 (p: 46.213/29.605, r: 100.0/100.0 when Conv1D/LSTM were used). This could be because typical motion aspects (for example, swinging the left arm slowly due to a heavy load in the left hand) are invariant to right-hand motion. Figure 13 exhibits example input signals with attention weights aligned when our system misidentified C8 as C6 during the blind test. In contrast to Figure 10 and Figure 11, which show examples of when the recognition process was correct, attention weights are not routinely and densely formed in Figure 13.

### 5.4. Evaluation on Walking-Related Datasets

We compared the classification results to those of other publicly available datasets. First, we used the PAMAP2 dataset (Physical Activity Monitoring for Aging People 2) [55], which includes 12 daily physical activities measured by on-body sensors attached to three different body parts, the hand, chest, and ankle. This dataset, interestingly, contains walking-related activities, such as walking, running, Nordic walking, ascending/descending stairs, and vacuum-cleaning. To achieve a temporal resolution comparable to our dataset, we downsampled the PAMAP2 dataset from 100Hz to 50Hz. The data was segmented into 3 s fixed-width sliding windows with no overlap. We also created a hand-oriented subset (PAMAP2-hand) using measurements from a sensor attached to the hand.

Second, we used the SBHAR dataset (Smartphone-Based HAR dataset with Postural Transitions), which is a multivariate time series data from 30 participants ranging in age from 19 to 48 years [56]. This dataset includes six basic activities (walking, walking upstairs, walking downstairs, sitting, standing, and lying) and six postural transitions (standing-to-sitting, sitting-to-standing, sitting-to-lying, lying-to-sitting, standing-to-lying, lying-to-standing). A smartphone mounted on the participant’s waist served as an inertial motion-capture device, equipped with a triaxial accelerometer and a gyroscope operating at 50 Hz. For testing our approach with the SBHAR dataset, we segmented the measurements using a sliding window of 3 s, with 50% overlap.

Third, we used the Daphnet freezing of gait (DG) dataset [7], which consists of inertial measurements (i.e., acceleration) from 10 Parkinson’s disease (PD) patients who are experiencing freezing of gait (FoG), which manifests as a sudden and temporary inability to move. The DG dataset is collected while PD patients are walking using on-body sensors attached to 3 different body parts (ankle, knee, and trunk). We validated our approach by downsampling our DG dataset from 66Hz to 50Hz and segmenting it with a sliding window of 3 s without overlap. Table 8 contains detailed information used for the evaluation. For more information on each dataset, see previous studies [10,57], which extensively summarizes the public dataset.

Table 8 shows performance in terms of weighted F1 scores (i.e., $F_{m}$) from the different public datasets along with ours. As shown below, we demonstrate that it is feasible to learn features from the walking-related activities, each of which is inherently bound to have similar temporal features, using the recent deep learning-based approaches. Although there is no significant performance improvements with the addition of attention, it enhances the explainability of the classification process.

## 6. Limitations and Applications

### 6.1. Limitations

The proposed model demonstrated high accuracies and F*_m_* in recognizing activities on the test set. However, as discussed in Section 4.2, it is not guaranteed that similar recognition performance can be achieved in real-life scenarios because our data was collected in a controlled environment and from a limited number of participants. In fact, Bao and Intille [13] emphasized the importance of unsupervised and naturally collected data. They collected two different types of data. One type was collected in a semi-naturalistic environment, wherein the participants were asked to complete descriptive tasks. This setting allowed participants to move on their own to some extent. The other type was collected in a laboratory setting where the participants were instructed to execute several predefined activities. Since our data was also collected in a controlled setting, we plan to design experiments in which participants are allowed to move more naturally.

### 6.2. Applications

Fine-grained classification of walking styles would open a new venue for promising applications in diverse fields, such as providing contextual information tailored to a user’s current situation, measuring precise energy expenditures during exercise, and monitoring abnormal activities.

*Assistance for Distracted Walkers*

As smartphones become more common, people often look at their smartphone screens, even when walking. Consequently, a distracted walker may get into an accident. A part of our study, i.e., differentiating walking activities while looking at the smartphone screen (C4/C5) from regular walking (C0), can be utilized to help walkers. For example, wearable assistants based on our approach could provide distracted walkers with warnings when they enter a busy street. Identifying or recognizing cognitive loads while walking using wearable devices would be interesting future work.

*Contextual Applications*

Furthermore, recognition of the availability of the user’s hands, e.g., walking with dumbbells in both hands (C8) and walking with an umbrella in either hand (C2/C3), would be useful for those who cannot use their hands to manipulate smart devices. For example, wearable applications could read incoming messages or open car doors automatically if the system recognized that a user was moving with luggage in both hands.

*Encouraging Fitness*

Recognition of fundamental activities, such as running and walking, are already embedded in modern consumer smartwatches. For example, the device may encourage us to stand up if we sit still for a long time, and the device can recognize whether we are walking or running for fitness.

As we investigated throughout this study, our activities could be recognized in much finer detail. A wearable system may encourage users who are exercising to walk faster if they are walking slowly or with their hands in their pockets. Without loss of generality, the proposed approach can be extended to summarize the recorded activities into a set of fine-grained activities, enabling personalized fitness suggestions and encouragements.

## 7. Conclusions

Assuming that hand motions are an important part of human walking activities and thus have different spatiotemporal characteristics according to the walking styles, we propose a wearable system that can recognize fine-grained walking patterns. To that end, we defined 18 different everyday walking styles and developed a wearable system that can capture a user’s body motions from their hand motion in the form of MTS signals. Then, we employed a set of machine-learning algorithms, including feature-based algorithms and recent deep-learning algorithms to learn the MTS data with the predefined walking patterns in a supervised fashion.

With our model, the LSTM-based approach demonstrated the best classification results in terms of accuracy (F*_m_*) of 97.158 (97.156). However, deep-learning-based approaches, including Conv1D, LSTM, GRU, LSTM + Att, and GRU + Att, generally exhibited higher classification performance, i.e., accuracy and F*_m_* greater than 95%. Despite of our extensive feature engineering work, feature-based approaches demonstrated poor classification performances overall. One remarkable finding from the experimental results was that walking activities with something in the dominant hand can be recognized even when the smartwatch is worn on the non-dominant side. Regarding the blind test, the classification results of accuracy (F*_m_*) were 87.290 (88.259) when Conv1D was employed. Our model has trouble robustly recognizing specific walking patterns, such as walking with something in the right hand and walking on inclined/stepped surfaces, according to the findings of the blind test. To resolve this generalization issue, we plan to collect more data on these activities from diverse users to make our pretrained model more robust. Using the attention-based neural networks, we further analyzed the classification results to understand the relative contributions of the MTS signals used in the classification process. In the application section, we explored a set of wearable applications that utilize the proposed fine-grained walking activity-recognition scheme. Future studies will focus on increasing the robustness of the model and extending the proposed approach to diverse healthcare applications.

## Figures and Tables

**Table 1:** Walking styles defined in our study.

No.	Class	Description
C0	walking	walking on flat ground
C1	walking snow	walking on thick snow (approx. 5 cm to 7 cm)
C2	walking umbrella left	walking with umbrella in left hand on a rainy day
C3	walking umbrella right	walking with umbrella in right hand on a rainy day
C4	walking phone left	walking while looking at a smartphone in the left hand
C5	walking phone right	walking while looking at a smartphone in the right hand
C6	walking dumbbell left	walking with a 2-kg dumbbell in the left hand
C7	walking dumbbell right	walking with a 2-kg dumbbell in the right hand
C8	walking dumbbell both	walking with a 2-kg dumbbell in each hand
C9	walking cross arms	walking with arms crossed
C10	walking hands behind back	walking with hands behind the back
C11	walking pocket both	walking with hands in pockets
C12	jogging	fitness running
C13	uphill	walking up a hill
C14	downhill	walking down a hill
C15	upstairs	walking up steps
C16	downstairs	walking down steps
C17	standing and sitting	doing something while standing or sitting

**Table 2:** List of studies on human activity recognition related to walking activities.

Type of Machine Learning	Classification Accuracy	Classification Model	Target Motions	Literature
Feature-Based	84.26%	decision tree	walking, walking carrying items, running, sitting, standing, lying, stretching, watching TV, scrubbing, folding laundry, brushing teeth, working, eating, or drinking, reading, bicycling, vacuuming, ascending stairs, riding elevator, or escalator	[13]
	90.80%	random forest	walking, running, sitting, lying	[3]
	93.30%	random forest	walking, running, ascending stairs, standing, sitting, kicking soccer ball, dribbling basketball, playing catch with tennis ball, typing, handwriting, clapping, brushing teeth, folding laundry, eating, or drinking	[4]
	93.91%	decision tree	walking, running, standing, walking slope-up, walking slope-down	[18]
	82.46%	Gaussian mixture model	walking, walking slope-up, walking slope-down, ascending stairs, descending stairs,	[16]
Deep Learning	99.24%	neural network (5-level layer)	walking, running, working, reading, studying, taking a rest, playing a computer game, eating, or drinking, cooking, washing dished, taking a transport	[1]
	91.06%	neural network (multilayer perceptron)	walking, running, ascending stairs, descending stairs, sitting, standing	[8]
	98.70%	neural network (multilayer perceptron)	walking, running, ascending stairs, descending stairs, sitting, standing, lying	[19]
	92.05%	neural network (multilayer perceptron)	walking, walking slope-up, walking slope-down, ascending stairs, descending stairs	[6]
	99.40%	neural network (CNN)	walking, running, jumping, ascending stairs, descending stairs, standing and sitting, lying, bicycling	[17]

**Table 3:** Durations of data acquired for the experiment.

No.	Class	Total Duration (min)	Total Duration (h)
C0	walking	188.7	3.14
C1	walking snow	109.2	1.82
C2	walking umbrella left	108.3	1.81
C3	walking umbrella right	86.8	1.45
C4	walking phone left	188.0	3.13
C5	walking phone right	188.0	3.13
C6	walking dumbbell left	180.2	3.00
C7	walking dumbbell right	191.8	3.20
C8	walking dumbbell both	70.5	1.18
C9	walking cross arms	99.8	1.66
C10	walking hands behind back	200.0	3.33
C11	walking pocket both	180.0	3.00
C12	jogging	96.2	1.60
C13	uphill	121.7	2.03
C14	downhill	148.2	2.47
C15	upstairs	194.2	3.24
C16	downstairs	174.0	2.90
C17	standing sitting	185.2	3.09
	Total	2768.8	46.45

**Table 4:** Features selected based on significance hypothesis test (a total of 180 features were used in this study).

Feature Name	Descriptions
sum_values	sum over the timeseries values
fft_coefficient	Fourier coefficients of the one-dimensional discrete Fourier transform for real input by fast Fourier transform algorithm
autocorrelation	autocorrelation coefficient
partial_autocorrelation	value of partial autocorrelation function at the given lag
ar_coefficient	unconditional maximum likelihood of an autoregressive process
fourier_entropy	binned entropy of the power spectral density of the time series
change_quantiles	average absolute value of consecutive changes of the time series inside the corridor
binned_entropy	binned entropy
agg_linear_trend	linear least-squares regression for values of the time series
permutation_entropy	permutation entropy
number_peaks	number of peaks of the time series
lempel_ziv_complexity	complexity estimate based on the Lempel–Ziv compression algorithm

**Table 5:** Accuracies and F*_m_* with respect to the machine learning models.

Approach	Model	T = 100 (Approx. 2 s)		T = 150 (Approx. 3 s)	
		Train Accuracy (F*_m_*)	Test Accuracy (F*_m_*)	Train Accuracy (F*_m_*)	Test Accuracy (F*_m_*)
Feature-based	NB	49.564 (48.494)	49.441 (48.442)	53.019 (51.801)	53.382 (52.108)
	RF	54.764 (49.016)	53.895 (48.152)	53.968 (48.096)	53.400 (47.439)
	SVM	88.513 (88.357)	83.524 (83.262)	88.843 (88.707)	84.933 (84.706)
Deep Learning	Conv1D	96.902 (96.893)	94.597 (94.571)	99.212 (99.211)	96.976 (96.971)
	GRU	99.968 (99.967)	96.122 (96.109)	100.0 (100.0)	96.788 (96.782)
	LSTM	99.970 (99.974)	96.252 (96.249)	99.989 (99.990)	97.158 (97.156)
	GRU + Att	99.949 (99.946)	96.157 (96.156)	99.997 (99.996)	96.902 (96.903)
	LSTM + Att	99.994 (99.996)	96.103 (96.091)	99.994 (99.993)	97.096 (97.097)

**Table 6:** The mean and standard deviation inference time per each MTS data ($\in {\mathbb{R}}^{T\times D}$). For the feature-based approach, elapsed time for the feature extraction is displayed in parentheses.

Approach	Model	T = 100 (Approx. 2 s) Inference Time (msec)	T = 150 (Approx. 3 s) Inference Time (msec)
Feature-based	NB	3.553 ± 0.327 (521.888 ± 3.797)	3.554 ± 0.092 (547.842 ± 0.479)
	RF	9.726 ± 0.908 (564.915 ± 3.968)	9.627 ± 0.448 (526.848 ± 9.573)
	SVM	8.947 ± 0.167 (519.796 ± 3.379)	7.249 ± 0.137 (566.064 ± 3.605)
Deep Learning	Conv1D	32.336 ± 2.131	31.831 ± 2.390
	GRU	34.947 ± 1.998	35.542 ± 2.173
	LSTM	34.493 ± 1.636	36.731 ± 2.022
	GRU + Att	35.261 ± 1.932	36.398 ± 1.934
	LSTM + Att	35.174 ± 1.908	36.693 ± 1.985

**Table 7:** Data acquired for the blind test with each duration displayed.

No.	Class	Duration (min)
C0	walking	2.4
C1	walking snow	2.1
C2	walking umbrella left	2.5
C3	walking umbrella right	2.0
C4	walking phone left	3.0
C5	walking phone right	3.0
C6	walking dumbbell left	1.9
C7	walking dumbbell right	2.1
C8	walking dumbbell both	2.9
C9	walking cross arms	2.1
C10	walking hands behind back	3.1
C11	walking pocket both	3.0
C12	jogging	1.4
C13	uphill	0.5
C14	downhill	0.7
C15	upstairs	0.7
C16	downstairs	0.5
C17	standing sitting	2.0
	Total	35.9

**Table 8:** Comparison of classification results in terms of weighted F1 scores. Here, we added bi-LSTM, which uses two LSTMs to learn sequential data based on both the past and the future context of the signals [58,59], for measuring performances.

	PAMAP2 [55]	PAMAP2-Hand [55]	SBHAR [56]	DG [7]	Our Dataset
Sensor	IMUs on the hand, chest and ankle	IMU on the hand	smartphone on the waist	embedded board on the waist	smartwatch on the wrist
Dim.	18 (=3 × 6)	6 (=1 × 6)	6 (=1 × 6)	9 (=3 × 3)	6 (=1 × 6)
# of classes	12 classes	12 classes	12 classes	2 classes	18 classes
Sampling rate	50 Hz (downsampled)	50 Hz (downsampled)	50 Hz	50 Hz (downsampled)	50 Hz
Window	3 s	3 s	3 s (50% overlap)	3 s	3 s
Metric	F*_m_*	F*_m_*	F*_m_*	F1	F*_m_*
LSTM+Att	92.850	86.830	94.123	82.596	97.097
GRU+Att	86.981	86.981	94.069	82.979	96.875
LSTM	91.383	86.188	93.353	82.805	97.156
bi-LSTM	92.112	84.805	93.132	82.756	97.122
Conv1D	93.093	89.811	94.295	85.272	96.971

## Data Availability

We cited the details of each dataset in the document.

## References

[1] Kwon M.C., Ju M., Choi S. Classification of various daily behaviors using deep learning and smart watch. Proceedings of the 2017 Ninth International Conference on Ubiquitous and Future Networks (ICUFN).

[2] Balli S., Sağbaş E.A., Peker M. (2019). Human activity recognition from smart watch sensor data using a hybrid of principal component analysis and random forest algorithm. Meas. Control.

[3] Fuller D., Anaraki J.R., Simango B., Rayner M., Dorani F., Bozorgi A., Luan H., A Basset F. (2021). Predicting lying, sitting, walking and running using Apple Watch and Fitbit data. BMJ Open Sport Exerc. Med..

[4] Weiss G.M., Timko J.L., Gallagher C.M., Yoneda K., Schreiber A.J. Smartwatch-based activity recognition: A machine learning approach. Proceedings of the 2016 IEEE-EMBS International Conference on Biomedical and Health Informatics (BHI).

[5] Laput G., Harrison C. Sensing Fine-grained hand activity with smartwatches. Proceedings of the 2019 CHI Conference on Human Factors in Computing Systems.

[6] Wang N., Ambikairajah E., Lovell N.H., Celler B.G. Accelerometry based classification of walking patterns using time-frequency analysis. Proceedings of the 29th Annual International Conference of the IEEE Engineering in Medicine and Biology Society.

[7] Bachlin M., Plotnik M., Roggen D., Maidan I., Hausdorff J.M., Giladi N., Troster G. (2009). Wearable assistant for Parkinson’s disease patients with the freezing of gait symptom. IEEE Trans. Inf. Technol. Biomed..

[8] Kwapisz J.R., Weiss G., Moore S.A. (2011). Activity recognition using cell phone accelerometers. ACM SIGKDD Explor. Newsl..

[9] Christ M., Braun N., Neuffer J., Kempa-Liehr A.W. (2018). Time series feature extraction on basis of scalable hypothesis tests (tsfresh–a python package). Neurocomputing.

[10] Alsheikh M.A., Selim A., Niyato D., Doyle L., Lin S., Tan H.P. Deep activity recognition models with triaxial accelerometers. Proceedings of the Workshops at the Thirtieth AAAI Conference on Artificial Intelligence.

[11] Hausdorff J.M., Edelberg H.K., Mitchell S.L., Goldberger A.L., Wei J.Y. (1997). Increased gait unsteadiness in community-dwelling elderly fallers. Arch. Phys. Med. Rehabil..

[12] Saito K., Zecca M., Sessa S., Lin Z., Bartolomeo L., Cosentino S., Petersen K., Ishii H., Ikai T., Takanishi A. Assessment of walking quality by using inertial measurement units. Proceedings of the 2012 First International Conference on Innovative Engineering Systems.

[13] Bao L., Intille S.S., Ferscha A., Mattern F. (2004). Activity recognition from user-annotated acceleration data. Pervasive Computing.

[14] Bui D.T., Nguyen N.D., Jeong G.-M. (2018). A robust step detection algorithm and walking distance estimation based on daily wrist activity recognition using a smart band. Sensors.

[15] Wang J.-S., Lin C.-W., Yang Y.-T.C., Ho Y.-J. (2012). Walking pattern classification and walking distance estimation algorithms using gait phase information. IEEE Trans. Biomed. Eng..

[16] Wang N., Redmond S.J., Ambikairajah E., Celler B.G., Lovell N.H. (2010). Can triaxial accelerometry accurately recognize inclined walking terrains?. IEEE Trans. Biomed. Eng..

[17] Alemayoh T., Lee J., Okamoto S. (2021). New Sensor data structuring for deeper feature extraction in human activity recognition. Sensors.

[18] Hanai Y., Nishimura J., Kuroda T. Haar-like filtering for human activity recognition using 3d accelerometer. Proceedings of the 2009 IEEE 13th digital Signal Processing Workshop and 5th IEEE signal Processing Education Workshop.

[19] Lockhart J.W., Weiss G.M. The benefits of personalized smartphone-based activity recognition models. Proceedings of the SIAM international Conference On Data Mining.

[20] Ordóñez F.J., Roggen D. (2016). Deep convolutional and lstm recurrent neural networks for multimodal wearable activity recognition. Sensors.

[21] Mekruksavanich S., Hnoohom N., Jitpattanakul A. Smartwatch-based sitting detection with human activity recognition for office workers syndrome. Proceedings of the 2018 International ECTI Northern Section Conference on Electrical, Electronics, Computer and Telecommunications Engineering (ECTI-NCON).

[22] Mekruksavanich S., Jitpattanakul A. Smartwatch-based human activity recognition using hybrid lstm network. Proceedings of the 2020 IEEE Sensors.

[23] Sun K., Xiao B., Liu D., Wang J. Deep high-resolution representation learning for human pose estimation. Proceedings of the IEEE/CVF Conference on Computer Vision and Pattern Recognition.

[24] Benjamini Y., Yekutieli D. (2001). The control of the false discovery rate in multiple testing under dependency. Ann. Stat..

[25] Cortes C., Vapnik V. (1995). Support-vector networks. Mach. Learn..

[26] Liaw A., Wiener M. (2002). Classification and regression by randomForest. R News.

[27] Bishop C.M. (2006). Pattern Recognition and Machine Learning.

[28] Gislason P.O., Benediktsson J.A., Sveinsson J.R. (2006). Random forests for land cover classification. Pattern Recognit. Lett..

[29] Kang G., Kim S.-C. (2020). DeepEcho: Echoacoustic recognition of materials using returning echoes with deep neural networks. IEEE Trans. Emerg. Top. Comput..

[30] Tran D., Bourdev L., Fergus R., Torresani L., Paluri M. Learning spatiotemporal features with 3d convolutional networks. Proceedings of the IEEE International Conference on Computer Vision.

[31] LeCun Y., Bengio Y. (1995). Convolutional networks for images, speech, and time series. The Handbook of Brain Theory and Neural Networks.

[32] Kim Y. Convolutional neural networks for sentence classification. Proceedings of the Conference on Empirical Methods in Natural Language Processing (EMNLP).

[33] Kiranyaz S., Avci O., Abdeljaber O., Ince T., Gabbouj M., Inman D.J. (2020). 1D convolutional neural networks and applications: A survey. Mech. Syst. Signal Process..

[34] Perol T., Gharbi M., Denolle M. (2018). Convolutional neural network for earthquake detection and location. Sci. Adv..

[35] Ryu S., Kim S.-C. (2020). Embedded identification of surface based on multirate sensor fusion with deep neural network. IEEE Embed. Syst. Lett..

[36] Han B.-K., Ryu J.-K., Kim S.-C. (2019). Context-aware winter sports based on multivariate sequence learning. Sensors.

[37] Kiranyaz S., Ince T., Hamila R., Gabbouj M. Convolutional neural networks for patient-specific ECG classification. Proceedings of the 37th Annual International Conference of the IEEE Engineering in Medicine and Biology Society (EMBC).

[38] Ryu S., Kim S.-C. (2020). Knocking and listening: Learning mechanical impulse response for understanding surface characteristics. Sensors.

[39] Hochreiter S., Schmidhuber J. (1997). Long short-term memory. Neural Comput..

[40] Chung J., Gulcehre C., Cho K., Bengio Y. (2014). Empirical evaluation of gated recurrent neural networks on sequence modeling. arXiv.

[41] Cho K., Van Merrienboer B., Gulcehre C., Bahdanau D., Bougares F., Schwenk H., Bengio Y. (2014). Learning phrase representations using RNN encoder-decoder for statistical machine translation. arXiv.

[42] Han B.-K., Kim S.-C., Kwon N.-S. (2018). DeepSnake: Sequence learning of joint torques using a gated recurrent neural network. IEEE Access.

[43] Dua N., Singh S.N., Semwal V.B. (2021). Multi-input CNN-GRU based human activity recognition using wearable sensors. Computing.

[44] Graves A., Mohamed A.-R., Hinton G. Speech recognition with deep recurrent neural networks. Proceedings of the IEEE International Conference on Acoustics, Speech and Signal Processing.

[45] Bahdanau D., Cho K., Bengio Y. (2014). Neural machine translation by jointly learning to align and translate. arXiv.

[46] Luong T., Pham H., Manning C. (2015). Effective approaches to attention-based neural machine translation. arXiv.

[47] Xu K., Ba J., Kiros R., Cho K., Courville A., Salakhudinov R., Zemel R., Bengio Y. Show, attend and tell: Neural image caption generation with visual attention. Proceedings of the International Conference on Machine Learning.

[48] Ran X., Shan Z., Fang Y., Lin C. (2019). An LSTM-based method with attention mechanism for travel time prediction. Sensors.

[49] Zeng M., Gao H., Yu T., Mengshoel O.J., Langseth H., Lane I., Liu X. Understanding and improving recurrent networks for human activity recognition by continuous attention. Proceedings of the 2018 ACM International Symposium on Wearable Computers.

[50] Kingma D.P., Ba J. (2014). Adam: A method for stochastic optimization. arXiv.

[51] Maaten L.v.d., Hinton G. (2008). Visualizing data using t-SNE. J. Mach. Learn. Res..

[52] Haque M.N., Mahbub M., Tarek M.H., Lota L.N., Ali A.A. Nurse care activity recognition: A GRU-based approach with attention mechanism. Proceedings of the 2019 ACM International Joint Conference on Pervasive and Ubiquitous Computing and In Proceedings of the 2019 ACM International Symposium on Wearable Computers.

[53] Ma H., Li W., Zhang X., Gao S., Lu S. AttnSense: Multi-level attention mechanism for multimodal human activity recognition. Proceedings of the Twenty-Eighth International Joint Conference on Artificial Intelligence.

[54] Gao W., Zhang L., Teng Q., He J., Wu H.D. (2021). DanHAR: Dual attention network for multimodal human activity recognition using wearable sensors. Appl. Soft Comput..

[55] Reiss A., Stricker D. Introducing a new benchmarked dataset for activity monitoring. Proceedings of the 2012 16th International Symposium On Wearable Computers.

[56] Anguita D., Ghio A., Oneto L., Parra X., Reyes-Ortiz J.L. A public domain dataset for human activity recognition using smartphones. Proceedings of the European Symposium on Artificial Neural Networks, Computational Intelligence and Machine Learning.

[57] Reyes-Ortiz J.-L., Oneto L., Sama A., Parra X., Anguita D. (2016). Transition-aware human activity recognition using smartphones. Neurocomputing.

[58] Chen T., Xu R., He Y., Wang X. (2017). Improving sentiment analysis via sentence type classification using BiLSTM-CRF and CNN. Expert Syst. Appl..

[59] Schuster M., Paliwal K.K. (1997). Bidirectional recurrent neural networks. IEEE Trans. Signal Process..

